# Epigenetics of cognition and behavior: insights from Mendelian disorders of epigenetic machinery

**DOI:** 10.1186/s11689-023-09482-0

**Published:** 2023-05-27

**Authors:** Rowena Ng, Allison Kalinousky, Jacqueline Harris

**Affiliations:** 1 Kennedy Krieger Institute, Baltimore, MD USA; 2 Department of Psychiatry and Behavioral Sciences, Johns Hopkins University School of Medicine, Baltimore, MD USA; 3 McKusick-Nathans Department of Genetic Medicine, Johns Hopkins University School of Medicine, Baltimore, MD USA; 4 Department of Pediatrics, Johns Hopkins University School of Medicine, Baltimore, MD USA; 5 Department of Neurology, Johns Hopkins University School of Medicine, Baltimore, MD USA

**Keywords:** Epigenetics, Neurodevelopment, Cognition, Behavior

## Abstract

Epigenetics, one mechanism by which gene expression can change without any changes to the DNA sequence, was described nearly a century ago. However, the importance of epigenetic processes to neurodevelopment and higher order neurological functions like cognition and behavior is only now being realized. A group of disorders known as the Mendelian disorders of the epigenetic machinery are caused by the altered function of epigenetic machinery proteins, which consequently affects downstream expression of many genes. These disorders almost universally have cognitive dysfunction and behavioral issues as core features. Here, we review what is known about the neurodevelopmental phenotypes of some key examples of these disorders divided into categories based on the underlying function of the affected protein. Understanding these Mendelian disorders of the epigenetic machinery can illuminate the role of epigenetic regulation in typical brain function and can lead to future therapies and better management for a host of neurodevelopmental and neuropsychological disorders.

## Background

In 1942, Conrad Waddington coined the term “epigenetics” to describe biological events that occur in response to external factors, therefore providing a relationship between genotype and phenotype [1]. Today, it is known that epigenetic changes are heritable modifications that alter gene expression patterns without changing the DNA sequence. This is done through DNA methylation, histone modifications, chromatin remodeling, and RNA-based mechanisms [2]. Together, these mechanisms are crucial for a wide range of processes, including early development, X-inactivation, imprinting, and tissue-specific gene regulation, which, if disturbed, can lead to detrimental consequences. Here, we will focus on the role epigenetics plays in neurodevelopment, specifically utilizing Mendelian disorders of the epigenetic machinery (MDEMs) as examples to explain the underpinnings of the consequences of dysregulation.

### Epigenetics in neurodevelopment

When examining the genes that have been found to be causative of neurodevelopmental disabilities (NDDs), several patterns emerge. Many of the genes share common pathways including neurogenesis—developmental and adult, proliferation and differentiation of neural progenitors, neural migration, axonal guidance, disrupted synaptogenesis, impaired synaptic function, and imbalance of excitatory and inhibitory signaling [3, 4]. Epigenetic regulation and chromatin modification play a crucial role in all aspects of these processes.

The most common type of DNA methylation occurs at the fifth position of cytosine at CpG dinucleotides and is traditionally associated with repression of gene expression at promoters, although several studies have suggested it can also be involved in upregulation of expression [5]. The regulation of this process is vital for neurodevelopment as suggested by the fact that certain brain regions and cell types have specific methylation signatures and that these methylation profiles start in flux and become fixed over the course of development [6, 7]. Additionally, non-CpG methylation is specifically enriched in brain tissues and accumulates throughout development into adulthood and has been shown to be crucial for both neurogenesis and maturation of neural progenitor cells [8]. DNA methylation is also the mechanism by which genes are imprinted. Imprinting is the process by which only one allele of certain autosomal genes is expressed in the body or in a certain tissue depending on the allele’s parent of origin. Imprinting disorders occur when the expression of imprinted genes are disrupted by a sequence change including deletion or duplication or epigenetic regulation. While imprinting disorders are outside the scope of this review, the existing literature on NDDs with disrupted genomic imprinting—like Prader-Willi syndrome and Angelman syndrome—highlight the vital role of imprinting on the regulation of gene expression, and subsequent, downstream effects on cognitive and behavioral functioning [9–11].

In addition to DNA methylation, 3D chromatin structure and the dynamic process of chromatin remodeling is a crucial piece underlying the transition of cells from neural progenitor cells (NPCs) to their terminally differentiated cell types. Along this differentiation process, certain regions of chromatin become increasingly more compressed causing certain genes—presumably those involved in dictating NPC fate—to become permanently repressed [12, 13]. Specific chromatin remodeler proteins have been shown to be essential for proper brain development and neuronal differentiation. For example, heterozygous knockout of the chromatin remodeler Chd2 in mice leads to a distinct deficiency in production of GABAergic neurons specifically, significantly altering the cellular makeup of the cortex [14]. Knockout of Ctcf in mice, one of the main proteins responsible for 3D chromatin organization, led to increased apoptosis and disorganization of the forebrain and telencephalon [15]. Importantly, in humans, heterozygous loss of function of CTCF and CHD2 are both associated with neurodevelopmental disorders resulting in intellectual disability, seizures, and behavioral issues [16].

Histone modifications also play an essential role in the development and functioning of the nervous system. The idea of histone modifications leading to either “open” or “closed” chromatin has long been established [17]. “Open” chromatin, or euchromatin, allows for transcription, resulting in gene expression, whereas “closed” chromatin, or heterochromatin, is more compact, making it difficult for factors to bind, resulting in silenced gene expression. As such, disruption of methylation or demethylation and/or acetylation or deacetylation at a variety of spots leads to abnormalities in neurodevelopment. For example, deletion of Dpy30—a common protein subunit among many histone methyltransferases—in mouse brains leads to both neurogenic and gliogenic deficits [18]. The polycomb repressive complex (PRC), one of the best studied mechanisms of gene expression regulation, contains both histone methyltransferase and histone demethylase components. Ezh2, a histone methyltransferase component of PRC2, has been shown to stimulate neurogenesis and stimulate proper differentiation of NPCs into neurons and glia [19, 20]. Maintaining the appropriate balance of the state of histone acetylation also seems to be particularly important for differentiation of NPCs into terminal cell types. Several studies have shown that an orchestrated and tightly regulated decrease and then increase of H3K9 acetylation is necessary for early neural differentiation, and if this process is disrupted, differentiation is either inhibited altogether or mistimed [21, 22].

While we continue to learn more about the role of epigenetics in neurodevelopment, some of the best evidence available comes from examining the resulting cognitive and behavioral phenotype when one specific component of the epigenetic machinery is disrupted. These disorders are collectively referred to as Mendelian disorders of the epigenetic machinery [23].

### Mendelian disorders of the epigenetic machinery (MDEMs)

The majority of MDEMs are caused by heterozygous loss of function variants in components of the machinery that perform the writing, erasing, reading, and remodeling of epigenetic marks [23]. Writers place the appropriate chemical groups, with the most widely studied alterations being methylation and acetylation. Methylation, or the addition of methyl group(s), can occur on both DNA, catalyzed by DNA methyltransferases (DNMTs) and typically associated with silencing of gene expression, and on histone proteins, catalyzed by histone methyltransferases (i.e., protein arginine methyltransferases (PRMTs) and histone lysine methyltransferases (KMTs)) which can result in open or closed chromatin. Acetylation, which occurs only on histones, is the addition of acetyl groups by histone acetyltransferases (HATs) and is typically associated with transcriptional activation [24]. Erasers, on the other hand, remove these modifications added to DNA or histones. Erasers include ten eleven translocation (TET) enzymes, which oxidize 5-methylcytosine to 5-hydroxymethylcytosine resulting in subsequent DNA demethylation, histone demethylases, which remove methyl groups from lysine residues in histones, and histone deacetylases (HDACs), which are enzymes that remove the acetyl group from histones [25].

These modifications then need to be recognized and complexes recruited, which is done by readers. Readers contain specialized docking domains which bind to specific covalent modifications placed by writers. For example, methyl CpG binding proteins (MBPs) bind to methylated DNA and recruit chromatin remodeling complexes to repress transcription [24]. The fourth type of epigenetic proteins are the remodelers. There are four structural families of chromatin remodelers including switch/sucrose non-fermenting (SWI/SNF), imitation switch (ISWI), inositol requiring 80-like (IN080), and chromodomain helicase DNA binding (CHD) [26]. These remodelers utilize adenosine triphosphate (ATP) to alter chromatin structures to either activate or repress gene expression.

It has become increasingly evident that variants causing alterations in the normal biallelic state of genes that encode proteins with direct roles in epigenetic regulation (www.epigeneticmachinery.org) often lead to disease. Many of these proteins that do cause disease when there is loss of function have dual epigenetic functions; for example, both an enzymatic and a reader domain [27]. Additionally, it has also been found that these genes that control epigenetic regulation have significantly higher probability of being loss of function intolerant (pLI) scores when compared to all other genes, indicating that these epigenetic machinery genes are highly intolerant to loss of function variation, with chromatin remodelers being the most highly intolerant group [27, 28]. This dosage sensitivity is an unusual feature of these genes, as about 80% of all Mendelian diseases caused by enzyme deficiency display recessive inheritance, whereas 86% of MDEMs display dominant inheritance [29, 30]. Interestingly, the vast majority of these MDEMs are associated with neurological dysfunction and more specifically, NDDs (Table 1) [23, 31]. This, along with the known role of epigenetics in neurodevelopment and ongoing neural circuitry functioning outlined above, underscores the importance of these mechanisms to the development of cognitive and behavioral phenotypes in humans in both health and disease. Several of the disease-causing histone modifiers have overlapping molecular mechanisms (e.g., writers at the same lysine residue, regulation of transcription of the same genes, etc.) [32]. Additionally, many of these proteins work together in complexes or are codependent [5]. If it is determined from deep phenotyping that MDEMs resulting from proteins with overlapping functions or codependent functions have similar cognitive or neurobehavioral endophenotypes, it gives us invaluable information about how these proteins and their epigenetic mechanisms function in both the developing and adult brains, not only in pathologic states but in health as well. Here, we will outline what is known about the cognitive and behavioral profiles of certain MDEMs divided into broad mechanistic categories to begin to examine these associations. Although literature involving cognitive and behavioral phenotypes of MDEMs are generally limited, the following MDEMs represent those with a relatively larger body of research or clinical case reviews that are also molecularly and/or phenotypically representative of the group.

**Table 1:** MDEM genes associated with neurodevelopmental disabilities and reported neurobehavioral phenotypes

Gene	Primary mechanism	Function	Dual function?	ID/DD	ADHD	Anxiety	Hypotonia	Other behavior problems
ALG13	Reader	HMR		X			X	Stereotypies, limited reports of SIB
ASH1L	Writer	HMT/HMR and HAR	X	X	X	X	X	ASD, SIB
ASXL1	Reader	HMR		X			X	
ASXL2	Reader	HMR		X	X		X	ASD
ASXL3	Reader	HMR		X			X	Stereotypies, autistic features, aggression
ATRX	Remodeler	RE/HMR	X	X	X		X	
BPTF	Reader	HMR and HAR		X		X	X	
BRPF1	Reader	HMR and HAR		X	X		X	
BRWD3	Reader	HMR and HAR		X			X	Aggression, autistic features
CHD1	Remodeler	RE/HMR	X	X			X	Autistic features
CHD2	Remodeler	RE/HMR	X	X	X			
CHD3	Remodeler	RE/HMR	X	X			X	Stereotypies
CHD4	Remodeler	RE/HMR	X	X				
CHD5	Remodeler	RE/HMR	X	X	X		X	Aggression, disruption
CHD7	Remodeler	RE/HMR	X	X			X	ASD, aggression, SIB, OCD
CHD8	Remodeler	RE/HMR	X	X	X	X	X	ASD, aggression, SIB
CREBBP	Writer	HAT/HAR	X	X	X	X	X	Repetitive behavior, aggression, SIB, OCD
DNMT3A	Writer	DNMT/HMR	X	X	X	X	X	ASD
DPF2	Reader	HAR		X			X	Behavioral prob NOS
EED	Reader	HMR		X			X	
EHMT1	Writer	HMT/HMR	X	X		X	X	ASD, aggression
EP300	Writer	HAT/HAR	X	X	X	X	X	Repetitive behavior, aggression, SIB, OCD
EZH2	Writer	HMT		X		X	X	ASD
HDAC4	Eraser	HDAC		X			X	
HDAC6	Eraser	HDAC		X				
HDAC8	Eraser	HDAC		X	X			ASD, aggression, SIB
KAT5	Writer	HAT		X	X			Stereotypies
KAT6A	Writer	HAT/HAR	X	X			X	
KAT6B	Writer	HAT/HAR	X	X			X	
KAT8	Writer	HAT		X	X		X	ASD
KDM1A	Eraser	HDM		X			X	
KDM3B	Eraser	HDM		X	X		X	ASD
KDM4B	Eraser	HDM/HMR	X	X	X		X	OCD
KDM5B	Eraser	HDM/HMR	X	X	X		X	
KDM5C	Eraser	HDM/HMR	X	X	X	X	X	Aggression, repetitive behaviors, stereotypies
KDM6A	Eraser	HDM		X	X	X	X	
KDM6B	Eraser	HDM		X	X		X	ASD
KMT2A	Writer	HMT/HMR and HAR and DNUMR	X	X	X	X	X	Repetitive behaviors, OCD, aggression
KMT2B	Writer	HMT/HMR and DNUMR	X	X	X		X	ASD, stereotypies
KMT2C	Writer	HMT/HMR	X	X	X		X	ASD, aggression
KMT2D	Writer	HMT/HMR	X	X	X	X	X	OCD, stereotypies
KMT2E	Writer	HMT/HMR	X	X	X	X	X	ASD, aggression, SIB, Stereotypies
KMT5B	Writer	HMT		X	X		X	ASD
MBD5	Reader	HMR and DNMR		X	X	X	X	ASD, Aggression, SIB, Stereotypies
MECP2	Reader	DNMR		X		X	X	ASD, aggression, SIB, repetitive behavior, stereotypies
MORC2	Reader	HMR		X			X	
MSL3	Reader	HMR		X			X	ASD
NSD1	Writer	HMT/HMR	X	X	X	X	X	ASD, aggression, repetitive behavior, OCD
PHF21A	Reader	HMR		X	X	X	X	ASD, SIB
PHF6	Reader	HMR		X			X	
PHF8	Eraser	HDM/HMR	X	X				ASD
PHIP	Reader	HMR and HAR		X	X	X	X	ASD, aggression, stereotypies
PRDM13	Writer	HMT		X				
PRDM8	Writer	HMT		X				
RAI1	Reader	HMR		X	X	X	X	Aggression, OCD
RERE	Reader	HMR		X			X	ASD, behavior problems NOS
SETD1A	Writer	HMT		X	X	X	X	Aggression, OCD
SETD1B	Writer	HMT		X		X		ASD
SETD2	Writer	HMT		X	X	X	X	ASD, aggression, OCD
SETD5	Writer	HMT		X		X	X	ASD, stereotypies, OCD
SMARCA2	Remodeler	RE/HAR	X	X				
SMARCA4	Remodeler	RE/HAR	X	X	X		X	Repetitive behaviors, stereotypies
SRCAP	Remodeler	RE		X	X	X	X	ASD, aggression, mood disorders
TCF20	Reader	HMR		X	X	X	X	ASD, mood disorders
TET3	Eraser	DNME		X	X	X	X	ASD, stereotypies
UBR7	Reader	HMR		X			X	
WHSC1 (NSD2)	Writer	HMT/HMR	X	X	X	X	X	ASD, stereotypies
ZMYND11	Reader	HMR and HAR		X	X		X	ASD, aggression

*ADHD* attention deficit hyperactivity disorder, *ASD* autism spectrum disorder, *DNME* DNA methylation eraser, *DNMR* DNA methylation reader, *DNMT* DNA methyltransferase, *HAR* histone acetyl group reader, *HAT* histone acetyltransferase, *HDM* histone demethylase, *HMR* histone methyl group reader, *HMT* histone methyltransferase, *ID/DD* intellectual disability/developmental disability, *OCD* obsessive compulsive disorder, *RE* chromatin remodeler, *SIB* self-injurious behavior

### *DNA methylation*—*Tatton-Brown-Rahman syndrome*

Tatton-Brown-syndrome (TBRS) is an overgrowth disorder caused by loss of function variants in *DNMT3A* which is involved in encoding an epigenetic regulator that mediates DNA methylation [33]. TBRS is characterized by intellectual impairment with the majority in the moderate range (IQ 39 to 76) [34, 35]. Recent literature suggests that those with TBRS may present with stronger verbal reasoning skills than non-verbal or spatial processing skills [35]. TBRS is associated with a high incidence of autism spectrum disorder (36–44%) [34, 35], and neuropsychiatric concerns, which includes psychotic disorders or schizophrenia largely varying from 5 to 42% of published clinical samples [34, 36]. Aggressive behaviors, stereotypic behaviors, obsessive compulsive behaviors, anxiety, features of attention-deficit/hyperactivity disorder, and neurodevelopmental regression have also been observed, although these may be associated with the broader clinical presentation of autism spectrum disorder or psychosis [34, 37]. Consistent with human subject research, mouse models with a heterozygous mutation in Dnmt3a similarly present with increased anxiety-related behaviors including reduced exploration and increased freezing response, as well as abnormal social behaviors (e.g., reduced exploration and communication with other mice) and reduced social drive, albeit, cognition was less affected [38].

### *DNA demethylation*—*Beck-Fahrner syndrome*

Beck-Fahrner syndrome (BEFAHRS) is the first identified Mendelian disorder of DNA demethylation caused by *TET3* deficiency [39]. Given the recent discovery of this syndrome, literature on the associated cognitive and behavioral phenotype is extremely limited. Of 11 cases of those with BEFAHRS or TET3 deficiency, all had global developmental delay or intellectual disability, most with hypotonia or hypermobility (9/11) and a little over half presented with autistic features (6/11) [39]. At present, the majority of the literature on TET3 deficiency has largely been conducted using animal models. Tet3 deficiency, deletion, or ablation in neurons have been linked to increased anxiety behaviors [40–42] and fear generalization in mice [42], in addition to impaired spatial orientation [41] and short-term memory [40], implicating its role in the neurogenesis of the hippocampus and prefrontal cortex. Moreover, expression of genes involved in memory formation are regulated by Tet3 levels [43]. Taken together, these findings may indicate hippocampal functions including spatial processing and semantic memory formation may be particularly affected among those with BEFAHRS, although this remains to be seen in human subject research. Anxiety and autistic traits, which may be bidirectionally related, may be more common features of the syndrome.

### *Chromatin remodeling—CHARGE syndrome*

The majority of those diagnosed with CHARGE syndrome have heterozygous mutations in CHD7 [44]. CHARGE syndrome is characterized by growth retardation in addition to multiple organ anomalies that includes the heart, choanae, genital/urinary systems, ear, and ocular coloboma, and, less commonly, vertebral/limb, renal, and tracheal abnormalities [44–46]. Cognitive functioning associated with CHARGE syndrome vary significantly with some studies reporting from impaired to broadly average intellect (IQ 54–92 in a case series of 7 patients) [47] albeit nearly half of those with the syndrome present with intellectual impairment (IQ < 70) in studies with larger samples (study with 50 participants) [48]. Hearing loss was represented in about a third of the sample which may contribute to speech/language delay [48]. Clinical studies involving patients with CHARGE syndrome report primary weaknesses in visuospatial construction, sequential processing, and selective attention juxtaposed with relative strengths in semantic skills, logical reasoning, and planning [47]. Notably, brain malformations, microcephaly, and low vision were prognostic predictors of intellectual functioning [49]. The neurobehavioral profile associated with CHARGE syndrome includes increased incidence of psychiatric illnesses including obsessive compulsive disorder (43–49%) and anxiety disorder (37–53%) [50, 51] with potential high comorbidity of the two psychopathologies [52]. Depression (8–24%) [51, 53, 54] and attention deficit hyperactivity disorder (ADHD) (26–34%) [52, 53], self-injurious behaviors (40–54%) [51, 53–55], and aggressive behaviors (38–53%) [51, 53, 54, 56] are also commonly found in those with this syndrome. Those with dual sensory impairment (deaf-blind) generally present with more challenging behaviors [53]. Autistic behaviors are commonly reported (26–43%) [50, 51, 53], although some research suggest these features may be due to sensory deficits, such as hearing loss and visual impairment, and are qualitatively different from the social impairment seen in idiopathic autism spectrum disorder [57].

### *Histone acetylation dysregulation*—*Rubinstein-Taybi syndrome and KAT6A syndrome*

Rubinstein-Taybi syndrome (RTS) and KAT6A syndrome are both neurogenetic disorders with variants in genes that regulate gene expression via histone acetylation. Heterozygous mutations in *CREBBP* or *EP300* genes—both involved in encoding transcription cofactors necessary for histone acetylation—cause RTS. KAT6A syndrome results from pathogenic variants in *KAT6A* which typically encodes lysine acetyltransferase and serves as a transcriptional coactivator. Despite shared disruptions in epigenetic machinery, emergent research suggests distinct cognitive and behavioral phenotypes across syndromes. While RTS is generally characterized by intellectual impairment and developmental delay, those with mutations in *EP300*, which represents a little less than 10% of cases [58], present with a milder form of intellectual disability than patients with variants in *CREBBP* [59]. Those with RTS have been described to show relative strengths in strong behavior regulation [60], social communication and affinity for interacting with others [61], regardless of severe intellectual impairment [62]. While limited studies have characterized cognitive functioning utilizing performance-based measures, the few extant investigations suggest verbal skills are relatively stronger than non-verbal and spatial skills among those with RTS [60, 63]. Behaviorally, attention problems, hyperactivity, and motor stereotypies are prevalent among those with RTS [61, 63]. Two studies report 37–43% of their samples with RTS met clinical cut-off for autism spectrum disorder utilizing screening questionnaires [64, 65]; however, given standardized measures were not used, these results may not reflect true prevalence of the developmental disorder. Externalizing problems are less consistently documented in research with RTS, whereas elevated rates of anxiety, obsessive compulsive disorder, and depression have been reported, particularly with older age [66].

In contrast, those with KAT6A syndrome uniformly show intellectual disability and developmental delay—with receptive/expressive language and communication deficits as the most well-documented feature [67]. Nearly 70% of affected individuals are minimally verbal [68]. Comprehension skills are reported to be more preserved than expressive language [67]. To date, with the exception of St. John et al. [68], descriptions of the syndrome have largely relied on retrospective review of medical history or case studies, and thus most non-verbal cognitive functions (e.g., non-verbal reasoning, spatial processing, executive functions, etc.) remains poorly characterized. Studies have documented 25–33% of those with KAT6A syndrome have a diagnosis of autism spectrum disorder although the rates may vary based on diagnostic methods applied [68]. Emotional disturbances appear to be less of a concern among affected individuals [69]. Heterogeneity in measurement tools combined with the absence of standardized neuropsychological assessments leave cross-MDEM comparisons challenging. Subsequent investigations may consider alternate methodologies (e.g., eye tracking) that can capture cognitive and behavioral processes across syndromes.

### *Histone methylation dysregulation*—*Kabuki syndrome and Wiedemann-Steiner syndrome*

Kabuki syndrome (KS) and Wiedemann-Steiner syndrome (WSS) are two MDEMS caused by pathogenic variants of a gene from the same KMT family of proteins. Most cases of KS result from heterozygous variants in *KMT2D* (80%) or *KMD6A* (5–10%) [70], and WSS is due to haploinsufficiency of *KMT2A* [71]. The *KMT* genes encode histone methyltransferases, thus, these disorders disrupt histone methylation and chromatin remodeling, with both KMT2D and KMT2A also having reader domains. Emergent evidence on the cognitive and neurobehavioral phenotypes of KS and WSS suggest some shared features. Intellectual functioning associated with KS ranges from severe impairment to average functioning although mild to moderate intellectual disability constitutes the majority of those affected [72–74]. Likewise, intellectual functioning estimates for those with WSS vary from moderate intellectual disability to average intelligence [75], with most in the mild to moderate impairment range [75, 76]. Interestingly, emergent evidence suggests some common characteristics in cognition across KS and WSS, with both sharing relative weaknesses in nonverbal reasoning, visuoconstruction, and visuospatial skills [74, 77–79], which implicate abnormal development of hippocampal formation as the potential underpinning of the shared pathogenesis of both MDEMs. Executive functioning and working memory are generally less affected in those with KS [77, 79], whereas recent case series examining cognitive functions in WSS suggest relative challenges in these areas [78]. KS is generally associated with few behavioral problems with anxiety, obsessive behaviors and attention difficulties most often observed by caregivers [80–82]. Social skills are considered a relative strength among those with KS, as individuals have been described as sweet and affectionate with relatively more preserved social adaptive skills [80, 83] paired with challenges in pragmatic language [83, 84]. Those with WSS present with elevated rates of anxiety [75, 78, 85] and behaviors concerning for ADHD [78, 85]. In contrast to the low levels of aggression seen in those with KS [80–82], relatively high rates of aggression [75] and conduct problems have been reported among those with WSS [85–87] which may be due to poorer executive functioning skills [85–87]. Rates of autism spectrum disorder in WSS vary widely across case studies given different clinical sample sizes and types of assessments used to determine diagnostic classification [75, 85, 88], although new evidence highlights similar high sociability and strong prosocial skills in those affected regardless of diagnosis of intellectual disability or autism spectrum disorder [88, 89]. In brief, KS and WSS are neurogenetic disorders with some common neuropsychological characteristics that may be reflective of shared disease-causing pathways which can be considered for future development of clinical trials.

## Conclusions

**Fig. 1:**
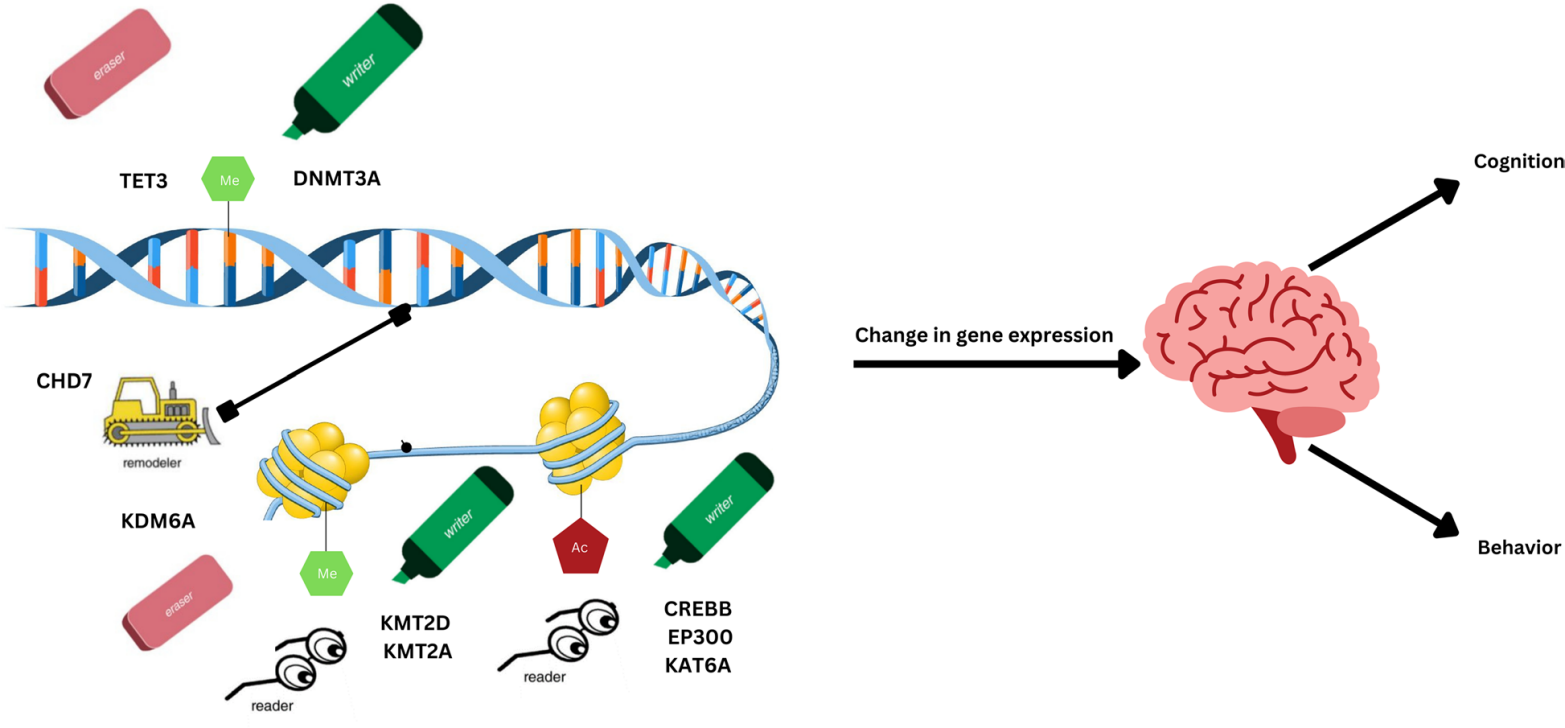
Epigenetic modifier proteins control cognition and behavior. An illustration summarizing the broad categories of epigenetic mechanisms that are disrupted in select MDEMs. Specifically highlighted are the mechanisms and conditions described in detail in this review. Mutations in epigenetic protein modifiers—DNA methylation writers (green marker), erasers (pink eraser), readers (glasses), and chromatin remodelers (truck)—drive atypical cognitive and behavioral development in MDEMs. Specifically, loss of function in different components of epigenetic machinery results in unique neurodevelopmental disorders. Variants in DNMT3A (a writer) affect DNA methylation causing Tatton Brown Rahman syndrome. Variants in TET3 (an eraser) affect DNA demethylation causing Beck Fahrner syndrome. Variants in CHD7 (a remodeler) impact chromatin remodeling causing CHARGE syndrome. Variants in CREBBP/EP300 and KAT6A (primary writers with a reader component) result in histone acetylation dysregulation causing Rubinstein Taybi syndrome and KAT6A syndrome, respectively. Variants in KMT2D (primary writer with reader component)/KDM6A (eraser) and KMT2A (primary writer with reader component) dysregulate histone methylation causing Kabuki syndrome and Wiedemann-Steiner syndrome, respectively

Mendelian disorders of the epigenetic machinery are a rapidly expanding group of disorders that almost universally have cognitive and behavioral issues as core features, and together account for a large proportion of genetic intellectual disability. We can start to understand the mechanisms of epigenetic control of cognition and behavior by not only examining similarities and differences in the cognitive and behavioral endophenotypes of MDEMs as an entire group, but also by dividing them into the epigenetic function of the protein translated from the causative gene, e.g., histone methyltransferases, chromatin remodelers, DNA methylation erasers, etc. (Fig. 1).

As science further elucidates the role of epigenetics in neurodevelopment, and more and more MDEMs are discovered that cause cognitive and behavioral issues, this area of study holds a great deal of promise not only for understanding how typical cognition and behavior is controlled but also for therapies for neurodevelopmental disabilities. One such area of promise is in the use of genome-wide DNA methylation “signatures". Most MDEMs as well as a number of other genetic NDD syndromes such as fragile X, have a very specific pattern of DNA methylation at certain identified regions of the genome that is extremely differentiated from unaffected individuals and from those with other genetic syndromes that it can be used to diagnose these disorders [90, 91]. While this alone is very exciting, even more intriguing is the idea that subtle variations within these syndrome-specific signatures may correlate with aspects of the cognitive and behavioral phenotype and can be used not only for diagnosis but also for prognosis and therapeutic monitoring [92]. In the future, larger sample sizes of individuals with MDEMs with known episignatures need to undergo systematic deep phenotyping combined with genome-wide DNA methylation analysis in order to identify correlations both within and between syndromes. This can also help to identify common signature loci and attributes for certain neurodevelopmental endophenotypes. Moreover, these findings and methodology may be applied to much more broad categories of neurodevelopmental and neuropsychiatric disorders.

Finally, this area of research involving MDEMs is likely to lead to novel and effective therapies for neurodevelopmental and neuropsychiatric disorders more broadly. While MDEMs are the example of the most direct and detrimental dysregulations of the epigenetic machinery, numerous other disorders from autism to epilepsy to schizophrenia have been linked to epigenetic machinery dysfunction [93–95]. Therapies used in these MDEMs to restore epigenetic balance may also prove very beneficial for a host of more common conditions. In effect, these rare genetic NDDs may be an important window to the understanding of the developmental processes of cognition and behavior in the human brain as well as possible treatment mechanisms to address disruptions in these pathways.

## Data Availability

Data sharing is not applicable to this article as no datasets were generated or analyzed during the current study.

## Abbreviations

ADHD: Attention deficit hyperactivity disorder
ATP: Adenosine triphosphate
BEFAHRS: Beck-Fahrner syndrome
CHD: Chromatin helicase domain
DNMT: DNA methyltransferase
HAT: Histone acetyltransferase
HDAC: Histone deacetylase
KMT: Lysine methyltransferase
KS: Kabuki syndrome
MBP: Methyl-binding protein
MDEM: Mendelian disorder of the epigenetic machinery
NDD: Neurodevelopmental disability
NPC: Neural progenitor cells
pLI: Probability of being loss of function intolerant
PRC: Polycomb repressor complex
PRMT: Protein arginine methyltransferase
RTS: Rubinstein-Taybi syndrome
TBRS: Tatton-Brown-Rahman syndrome
TET: Ten eleven translocation
WSS: Wiedemann-Steiner syndrome
IN080: Inositol requiring 80-like
ISWI: Imitation switch
SWI/SNF: Switch/sucrose non-fermenting
